# Laser Nanostructuring for Diffraction Grating Based Surface Plasmon-Resonance Sensors

**DOI:** 10.3390/nano11030591

**Published:** 2021-02-26

**Authors:** Iaroslav Gnilitskyi, Sergii V. Mamykin, Christina Lanara, Ihor Hevko, Mykhaylo Dusheyko, Stefano Bellucci, Emmanuel Stratakis

**Affiliations:** 1 NoviNano Lab LLC, 79015 Lviv, Ukraine; igorgevko96@gmail.com; 2 Department of Photonics, Lviv Polytechnic National University, 79021 Lviv, Ukraine; 3 V. E. Lashkaryov Institute of Semiconductor Physics, National Academy of Sciences of Ukraine, 02000 Kyiv, Ukraine; smamykin@gmail.com; 4 Foundation for Research and Technology-Hellas (F.O.R.T.H.), Institute of Electronic Structure and Laser (I.E.S.L.), 70013 Heraklion, Greece; lanarachris57@gmail.com (C.L.); stratak@iesl.forth.gr (E.S.); 5 Department of Materials Science and Technology, University of Crete, 70013 Heraklion, Greece; 6 Kiev Polytechnic Institute, National Technical University of Ukraine, 03056 Kyiv, Ukraine; mgd61@ukr.net; 7 INFN-Laboratori Nazionali di Frascati, 00044 Frascati, Italy; stefano.bellucci@lnf.infn.it

**Keywords:** ultrafast laser ablation, silicon, laser-induced periodic surface structures, HR-LIPSS, laser nanostructuring, femtosecond laser, surface plasmon resonance, plasmonic nanoparticles

## Abstract

The surface plasmon resonance properties of highly regular laser-induced periodic surface structures (HR-LIPSSs) on Si, functionalized with Au nanoparticles (NPs), were investigated. In particular, the spectral dependencies of polarized light reflectance at various angles of incidence were measured and discussed. It is found that the deposition of Au NPs on such periodically textured substrates leads to significant enhancement of the plasmon resonance properties, compared to that measured on planar ones. This effect can be used to improve the efficiency of localized-plasmon-resonance-based sensors.

## 1. Introduction

Many advanced optoelectronic devices contain periodically corrugated surface optical elements, i.e., phase diffraction gratings. Plasmon-active metals, which are placed on the respective gratings, like a silver or gold, enable the excitation of a few surface electromagnetic modes. Such modes can be applied as a basis for sensing and photovoltaic applications. For example, depositing metal films and loosely packed metal nanoparticles may enable excitation of surface plasmon polaritons (SPPs), whereas excitation of surface plasmon (SP) may be noticed in experiment with individual metal NPs [1,2]. Based on the respective SP [3] and SPP [4], sensors are already utilized to spot little changes in effective refractive index of the environment on account of various chemical or biological reactions [5]. For certain geometry, the simultaneous excitation of SP and SPP may appear [6]. Generally, the manufacturing of diffraction gratings via lithography processes takes a long time and is a complex and high-cost procedure.

A promising alternative can be via application of femtosecond lasers, due to their unique non-thermal material ablation property [7], that enables avoiding heat-induced damage [8]. Femtosecond pulses have also been exploited to induce grating-like periodic surface structures in various materials, such as metals. In principle, the scattered light from surface defects interfering with the incoming laser beam causes a periodic intensity pattern. Such an intensity pattern results in periodic surface structures known as laser-induced periodic surface structures (LIPSSs) [9]. Laser-induced periodic structures have been demonstrated on metals [10,11,12], semiconductors [13], dielectric surfaces [14], and polymers [15] and have been used in various applications [16], including hydrogen sensors [17], plasmonics [18], colorizing metals [19], wettability [20], and tribology [21] applications.

Most of the LIPPS fabrication methods reported to date lack the long-range ordered coverage required for large area applications, such as display technologies [22] and also the high degree of periodicity demanded by photonics and photovoltaics applications [23]. This is due to the absence of a feedback mechanism capable of guaranteeing the translational invariance of surface structures. Recently, highly regular LIPSSs (HR-LIPSSs) were developed via a low-cost single-step maskless process and industrially accepted speed production [24]. It is based on femtosecond laser superheating and ablation mechanism, enabling the coverage of large areas in many materials with highly uniform periodic structures [25]. In the present work, we report the creation of optically resonant photonic structures on Si substrate via the use of HR-LIPSS. The application of the fabricated HR-LIPSS in the enhancement of the surface plasmon resonance of Au NPs, which are chemically bonded to the laser textured Si gratings, is demonstrated and discussed.

## 2. Materials and Methods

### 2.1. Fabrication of HR-LIPSS on Si Substrates

For this work, single crystalline undoped Si <111> wafers with 4 Ohm·cm resistivity and 300 µm thickness were used.

**Figure 1:**
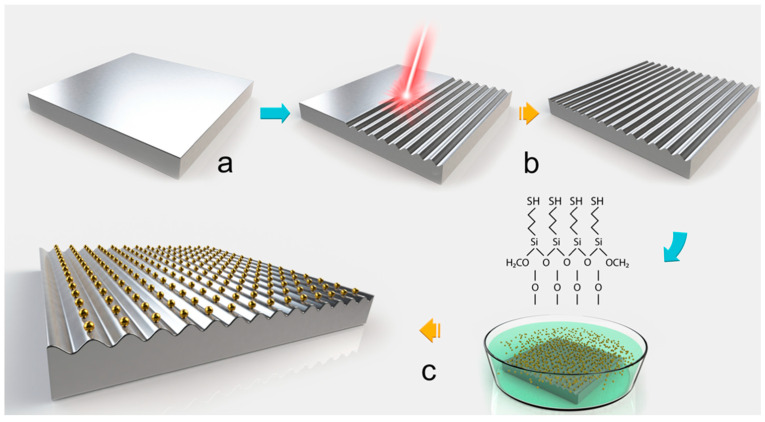
Schematic of the experimental process followed the fabrication of the Au nanoparticles (NPs)-based Si gratings. Flat Si substrate (**a**); nanopatterned by highly regular laser-induced periodic surface structure (HR-LIPSS) substrate of Si during ultrashort pulsed laser processing (**b**); functionalization procedure of nanopatterned Si surfaces and Au NPs immobilization procedure onto nanopatterned Si surfaces (**c**).

A schematic of the different steps required for the fabrication of Au NPs-based Si gratings is presented in Figure 1. For the fabrication of HR-LIPSS, Yb:KGW chirped-pulse application laser system (model PHAROS P-20 from Light Conversions Ltd.) was used. All experiments were carried out by using laser wavelength 1030 nm and pulse duration 266 fs. To accelerate the processing speed, a galvanometric scanning head (ScanLab) was applied. Alignment of linearly polarized laser light was controlled by a half-wave plate. The surface treatment was performed in air at room temperature by scanning laser beam over sample surface. The laser beam was focused by an F-theta lens with focal length 56 mm that produced an approximate diameter of the irradiation spot on the sample of 10.4 µm (1/e^2^ of peak intensity). At these parameters, the peak fluence on the surface was equal to ~0.76 J/cm^2^.

### 2.2. Deposition of Gold Nanoparticles onto the Nano-Structured Si Substrates

During the experiment, we thermally oxidized at 1000 °C for 30 min, in air, the flat and nanopatterned Si substrates. Such oxidization brings forth a conformal silicon oxide layer. After that, the substrates were activated by immersion in Piranha solution (i.e., H_2_SO_4_: H_2_O_2_ = 3:1 (*v*/*v*)), for 30 min, at room temperature (RT). Then we rinsed samples with nano-pure water and dried with nitrogen gas. Following the above stage, we dipped the substrates into MPTMS solution in dry toluene (1.85% (*v*/*v*)), for 3 h, at room temperature. Then, the substrates were gargled in toluene and ethanol (two times), and the procedure was completed by drying the substrates with nitrogen gas and thermal annealing at 100 °C for 30 min. It should be noted that, in order to bind the Au NPs onto the MPTMS-functionalized surfaces, we used the evaporation from solution. To reach that, a drop of 70 μL was placed onto the MPTMS-functionalized silicon surfaces, they were allowed to evaporate for 16 h, and then we rinsed the sample with nano-pure water.

### 2.3. Morphological Characterization

All the surfaces were morphologically characterized by scanning electron microscopy (SEM), on a JEOL 7000 field emission scanning electron nanoscope (FESEM; JEOL 7000) (JEOL (Europe) BV, Zaventem, Belgium), or a FEI Nova NanoSEM 450, Billerica, MA, USA). equipped with energy-dispersive X-ray spectroscopy (EDS), model Bruker QUANTAX-2 (Billerica, MA, USA).). In order to calculate the statistical parameters for the nanopatterned and Au NPs-functionalized substrates, as well as the Au NPs density (Table 1), an image-processing algorithm (ImageJ, National Institutes of Health, Bethesda, MD, USA) was implemented.

### 2.4. Optical Characterizations

Once the respective experiment was finalized, we researched the optical properties of the samples by applying measurements of spectral and angular dependence of P- and S-polarized light reflection in the 0.4–1.1 μm wavelength range and 10–70° angles of incidence. The halogen tungsten lamp, the mechanical light chopper, a monochromator with Glan prism at the exit, and a rotary table for samples were used for the measurements of the samples [2]. The light reflection intensity was measured by a silicon photodetector, the signal of which was applied to the input of analog-to-digital converter. The dimension possibilities of such a setup permitted us to construct the dispersion curves of excited optical modes and to determine their types.

## 3. Results and Discussiondone

### 3.1. Nanopatterned Silicon Substrates Decorated with Au NPs

**Figure 2:**
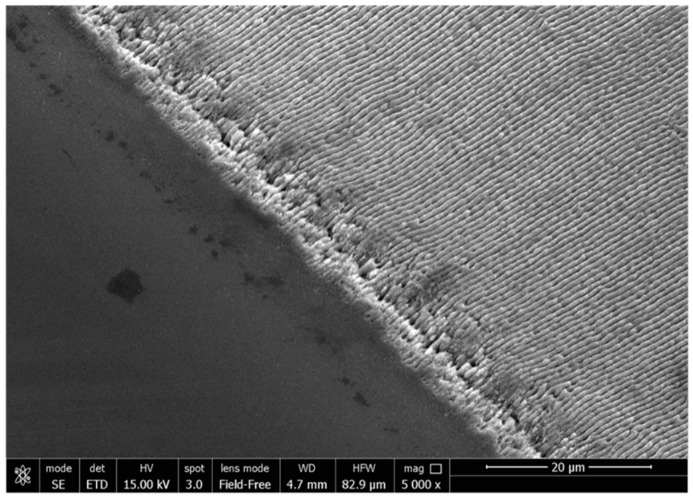
SEM image of flat (left) and HR-LIPSS generated on Si surface.

Typical SEM images of the laser-patterned Si surfaces, presented in Figure 2, show the formation of highly regular and homogeneous linear LIPSS with practically no bifurcations over the entire treated area (~10 μm wide). Nanoparticles observed onto the LIPSS indicate possible back-deposition of the ablation products.

**Figure 3:**
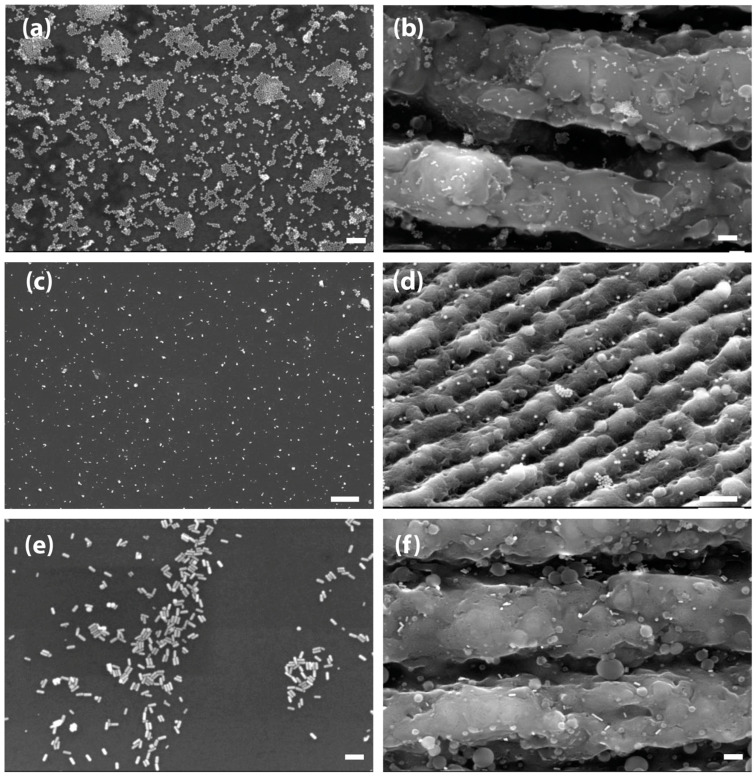
SEM images of spherical Au NPs with 13 nm size diameter, covalently bound onto flat (**a**) and nanopatterned Si substrates (**b**) (scale bars: 100 nm) diluted in 40% ethanol; spherical Au NPs with 80 nm diameter, covalently bound onto flat (**c**) and nanopatterned (**d**) Si substrates (scale bars: 1 µm) deposited via drop evaporation protocol diluted in 40% of ethanol; rod-shaped with 53 nm length Au NPs and aspect ratio 3:1, covalently bound onto flat (**e**) and nanopatterned (**f**) Si substrates (scale bar: 100 nm) deposited via drop evaporation protocol diluted in 40% of ethanol.

Following the Au NPs deposition protocol (Figure 1), the Au NPs were successfully attached to the surfaces, as confirmed by SEM imaging. Using the protocol of droplet evaporation after surface functionalization, a plethora of Au NPs with various shapes and sizes, including spheres and rods, were successfully deposited (Figure 3). Thus, sphere-shaped Au NPs with 13 nm diameter (Figure 3a,b), sphere-shaped Au NPs with 80 nm diameter (Figure 3c,d), and rod-shaped Au NPs with 53 nm length, aspect ratio 3:1 (Figure 3e,f), were chemically attached to both flat and nanopatterned Si substrates. Remarkably, the Au NPs could conformably disperse over the entire surface area of the nanostructured substrates, covering both the “ripples” (Figure 3b,d,f) and the surrounding flat area (Figure 3a,c,e). Au NPs were in the form of single particles but also in the form of small clusters (Figure 3c). Finally, to improve the sphere-shaped Au NPs’ (13 nm diameter) dispersion onto the functionalized surfaces, Au NPs solution was diluted in ethanol before the drop evaporation step.

**Figure 4:**
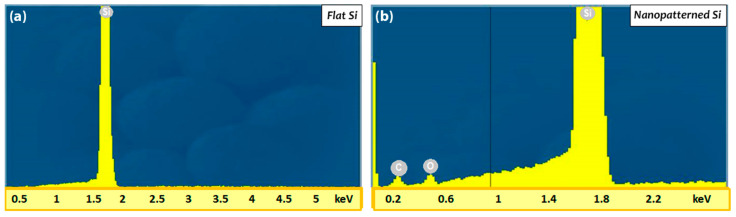
EDS spectrums of Si substrates: (**a**) untreated and (**b**) treated by HR-LIPSS Si substrates.

Elemental analysis with energy-dispersive X-ray spectroscopy (EDS) of the nanopatterned Si substrates showed that, apart from the Si peak (Figure 4b), carbon (C) and Oxygen (O) elements are observed in respect to untreated Si, where we observed the only peak of Si (Figure 4a).

### 3.2. Spectroscopy Results of the Hierarchical Nano-Patterned Si Substrates

**Figure 5:**
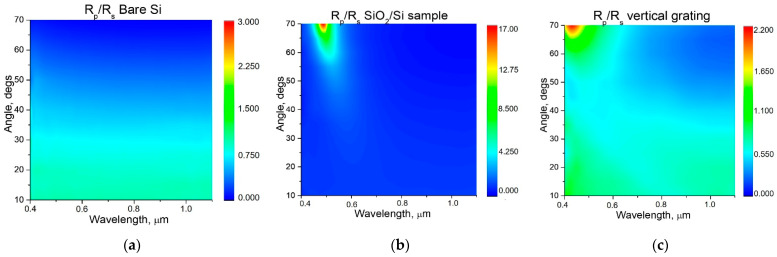
Map of R_p_/R_s_ ratio of light reflection for bare Si with native oxide (**a**), flat oxidized Si substrate (**b**), and oxidized Si with vertically oriented LIPSS diffraction gratings (**c**).

**Figure 6:**
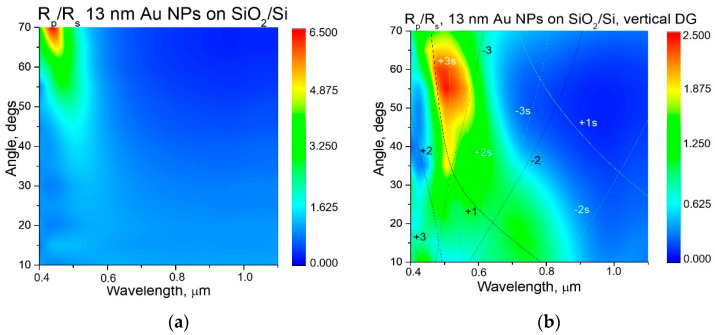
Comparison between samples with 13 nm Au NPs on oxidized flat Si (**a**) and on oxidized Si with vertically aligned diffraction grating (DG) (**b**). Dispersion curves (**b**) for surface plasmon-polariton waves for two boundaries, air/gold (±1, 2, and 3) and gold/silica (±1s, 2s, and 3s), are shown for different diffraction orders 1, 2, and 3 (shown on the figures).

**Figure 7:**
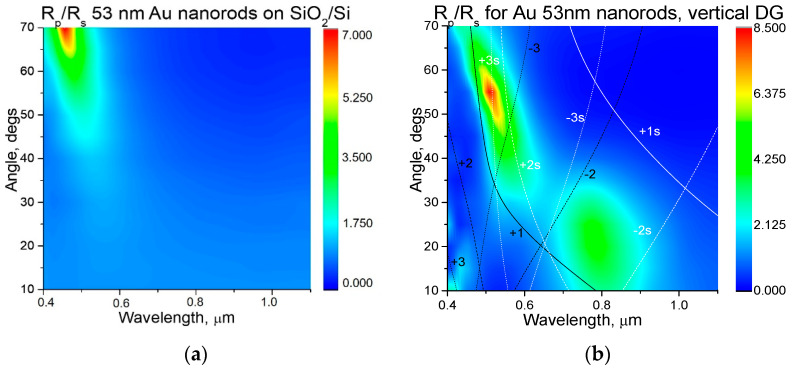
Comparison between samples with 53 nm Au nanorods on oxidized flat Si (**a**) and on oxidized Si with vertically aligned DG (**b**). Dispersion curves (**b**) for surface-plasmon-polariton waves for two boundaries, air/gold (±1, 2, and 3) and gold/silica (±1s, 2s, and 3s), are shown for different diffraction orders 1, 2, and 3 (shown on the figures).

The thickness of the SiO_2_ layer on the oxidized Si substrate was determined from reflectance measurements to be equal to 80, 93, and 94 nm for the samples with deposited Au NPs 13 nm, Au nanorods 53 nm, and Au NPs 80 nm respectively. To account for the optical properties of the different samples prepared, the spectra of light reflection R_p_, R_s_ at different incidence angles for samples with vertically oriented diffraction gratings (grating ripples perpendicular to light incidence plane), and for flat oxidized Si were measured for *p*- and *s*-polarizations. The corresponding maps of the R_p_/R_s_ ratio are presented on Figure 5, Figure 6 and Figure 7. On these maps, the dispersion curves for SPP waves for two boundaries, air/gold (±1, 2, and 3) and gold/silica (±1s, 2s, and 3s), are shown by lines for the different diffraction orders 1, 2, and 3 (shown on Figure 5. The spectra of the R_p_/R_s_ ratio enable us emphasize the effect of excitation of plasmonic modes in case they exist. Positions of SPP wave in coordinates of angle of light incidence can be calculated from the phase-matching condition [26,27]:

*k sinθ* + *m**G*** = *k*_PP_ (1)

where *k* = 2π/(*λ/n*)—wave vector of the incident radiation with a wavelength *λ* in a vacuum; *θ*—angle of incidence; *m*—an integer (*m*^1^0) and denotes the diffraction order; ***G*** = 2π/*a*—reciprocal vector of grating with a period of *a*, ε = *n*^2^—permittivity and refractive index of the environment; *k*_PP_—wave vector of SPP. The SPP wave vector is assumed to be the same as for a smooth metal film [2,26]:

*k*_PP_ = ±(2π/(*λ*/*n*)) [ε_Me_ε/(ε_Me_ + ε)]^1/2^ (2)

where *k*_PP_ have “+” sign at *m* > 0, and “-” at *m* < 0. Here ε_Me_ = ε′_Me_ + iε″_Me_ = (*n + ik*)^2^—complex permittivity of the metal at the wavelength *λ*. For calculations, the optical constants of gold from Reference [27] were used, and the refractive index of the substrate was taken equal to n_s_ = 1.48, i.e., that of SiO_2_. For a given gratings period and optical constants, the excitation of modes with *m* = +1 (1, 1s) and *m* = −1 (−1, −1s) are possible at the interface air/gold (1, −1) and gold/substrate (1s, −1s).

Light reflectance for flat oxidized Si substrate (Figure 5) measured as reference to show the effect of surface texturization complemented with Au NPs. As it can be seen, no special features are observed for bare Si, but for oxidized Si (flat and with diffraction grating), there is a strong maximum at 0.5 um and large angles 40–70°. This maximum is due to interference in thick SiO_2_ layer on Si substrate. For the sample with LIPSS diffraction grating (DG), this maximum is a little bit shifted to the blue range, due to a slightly higher SiO_2_ thickness of 100 nm (Figure 5c).

As shown in Figure 6, the deposition of 13 nm Au NPs does not influence the R_p_/R_s_ ratio for flat oxidized Si; however, for samples with DG, some additional features appeared. Firstly, the maximum around 0.5 µm at 40–70° becomes more intense. Secondly, there is an enhancement of the R_p_/R_s_ ratio along the dispersion curve corresponding to main +1 order of the SPP wave that appeared on the boundary with air (see +1 dispersion black solid on Figure 6b).

Figure 7 presents the maps of the R_p_/R_s_ ratio for oxidized Si samples with deposited Au nanorods. The effect of nanorods is almost not observed for flat Si samples (Figure 7a); there is only an R_p_/R_s_ maximum present, which is related to the interference with SiO_2_ layer, which is also observed for flat oxidized Si without Au NPs. Apart from this maximum, the samples with DG also show a maximum around 0.8 µm, 0–30° for vertical DG. Compared to samples with 13 nm Au NPs, the nanorods sample shows more pronounced resonance at 0.53 µm.

**Figure 8:**
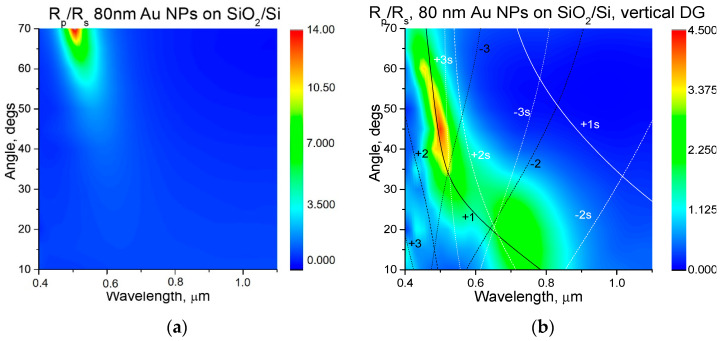
Comparison between samples with 80 nm Au NPs on oxidized flat Si (**a**) and on oxidized Si with vertically aligned DG. Dispersion curves (**b**) for surface-plasmon-polariton waves for two boundaries, air/gold (±1, 2, and 3) and gold/silica (±1s, 2s, and 3s), are shown for different diffraction orders 1, 2, and 3 (shown on the figures).

Figure 8 shows the R_p_/R_s_ ratio for oxidized Si samples with deposited 80 nm Au NPs. It can be observed that the flat oxidized Si with Au NPs do not exhibit any difference compared with sample without NPs; in particular, the same maximum at 0.5 µm, 50–70° is observed in both cases. For the vertically aligned DG two maxima are observed at 0.5 µm, 30–70° and 0.75 µm, 10–30°. This sample is found to be the most suitable for SPP wave excitation because the presence of larger Au NPs increases the range of near field interaction. The dispersion curve corresponding to main +1 order of SPP wave also fits well to R_p_/R_s_ maximum observed.

**Figure 9:**
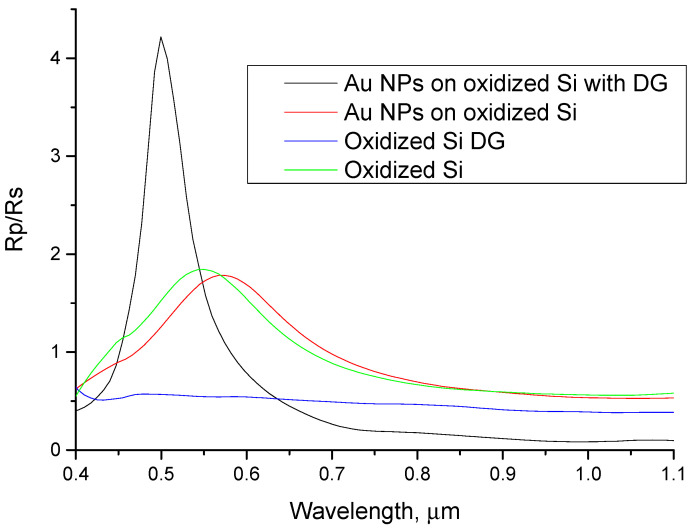
Comparison between samples with and without 80 nm Au NPs on oxidized flat Si and on oxidized Si with vertically aligned DG.

In conclusion, the LIPSS structures comprising the DG behave similarly to flat oxidized Si. As a consequence, the DG itself introduces a small effect into the respective optical spectra. On the contrary, the addition of plasmon-active metal NPs dramatically changes the nature of the reflection spectra. More important, this is not evident in the case of NPs deposited onto planar oxidized silicon. Figure 9 shows the reflectivity spectra R_p_/R_s_ for structures with flat and diffraction grating surface with and without Au NPs measured at a 45° angle of light incidence to emphasize the joint effect of Au NPs deposited on diffraction grating. As can be seen from the figure, the reflectivity spectrum of DG with deposited Au NPs has narrower and more intense resonance around 0.5 µm. Sensors based on surface plasmon resonance use the dependence of the spectral position of the resonance on the parameters of the environment. Therefore, it is obvious that their ability to measure resonance shift will depend on the acuity and intensity of that resonance. Thus, we believe that sensors based on gold nanoparticles on a diffraction grating will have a higher sensitivity. To optimize geometrical parameters of the considered structures, a theoretical model for Au NPs deposited on an oxidized Si substrate with diffraction grating will be developed in our future studies. It can therefore be concluded that this methodology can be employed to improve the efficiency of localized-plasmon-based sensors.

## 4. Conclusions

Highly regular LIPSS structures can be obtained on Si, which is suitable for the design of diffraction-grating-based SPR devices. It is shown that such LIPSS structures can be easily functionalized with Au NPs of different sizes giving rise to significant enhancement of the plasmon resonance features, compared to planar Si. This effect can be used to improve the efficiency of localized-plasmon-resonance-based sensors.

## Figures and Tables

**Table 1:** Au NPs’ density distribution on both flat and nanostructured Si substrates (per um^2^).

Au NPs Density (per μm^2^)		
Au NPs Shape	Flat Si Substrates	Nanostructured Si Substrates
Spheres 13 nm diameter	522	100
Spheres 80 nm diameter	14	25
Rods 3:1 aspect ratio	60	55

## Data Availability

Not applicable.
